# Optimization of prophylaxis for hemophilia A

**DOI:** 10.1371/journal.pone.0192783

**Published:** 2018-02-15

**Authors:** Robert D. Herbert, Carolyn R. Broderick, Chris Barnes, Laurent Billot, Albert Zhou, Jane Latimer

**Affiliations:** 1 Neuroscience Research Australia (NeuRA), Randwick, NSW, Australia; 2 University of New South Wales, Kensington, NSW, Australia; 3 The Children’s Hospital at Westmead, Westmead, NSW, Australia; 4 The Royal Children’s Hospital, Melbourne, VIC, Australia; 5 The George Institute for Global Health, Camperdown, NSW, Australia; 6 University of Sydney, Camperdown, NSW, Australia; University of Pennsylvania Perelman School of Medicine, UNITED STATES

## Abstract

**Background & aims:**

Prophylactic injections of factor VIII reduce the incidence of bleeds and slow the development of joint damage in people with hemophilia. The aim of this study was to identify optimal person-specific prophylaxis regimens for children with hemophilia A.

**Methods:**

Analytic and numerical methods were used to identify prophylaxis regimens which maximize the time for which plasma factor VIII concentrations exceed a threshold, maximize the lowest plasma factor VIII concentrations, and minimize risk of bleeds.

**Results:**

It was demonstrated analytically that, for any injection schedule, the regimen that maximizes the lowest factor VIII concentration involves sharing doses between injections so that all of the trough concentrations in a prophylaxis cycle are equal. Numerical methods were used to identify optimal prophylaxis schedules and explore the trade-offs between efficacy and acceptability of different prophylaxis regimens. The prophylaxis regimen which minimizes risk of bleeds depends on the person’s pattern of physical activity and may differ greatly from prophylaxis regimens that optimize pharmacokinetic parameters. Prophylaxis regimens which minimize risk of bleeds also differ from prophylaxis regimens that are typically prescribed. Predictions about which regimen is optimal are sensitive to estimates of the effects on risk of bleeds of factor VIII concentration and physical activity.

**Conclusion:**

The methods described here can be used to identify optimal, person-specific prophylaxis regimens for children with hemophilia A.

## Introduction

Prophylactic intravenous injections of factor VIII reduce the incidence of bleeds and slow the development of joint damage in children with severe hemophilia A [1–3].

Often prophylaxis regimens are designed with the objective of keeping plasma factor concentrations above 1 IU/dL [4]. This is because people with endogenous factor concentrations of less than 1 IU/dL typically display a severe disease phenotype [5]. An important observational study conducted by Collins and colleagues found that the incidence of bleeds in older children and adults with severe hemophilia A decreased by 1.4% for every hour of the week that factor VIII concentrations remained above 1 IU/dL.

There has been extensive discussion of how pharmacokinetic profiles can be used to develop personalised prophylaxis regimens [6]. It has been shown that the dose required to keep plasma factor VIII concentrations above 1 IU/dL is sensitive to the frequency of injections and factor half-life but not to the in vivo recovery [7].

While there has been extensive discussion of personalised prophylaxis there has not, to our knowledge, been a systematic effort to identify which of all possible prophylaxis regimens is optimal for an individual. We refer to the prophylaxis regimen which is the best of all possible prophylaxis regimens for a particular individual as the “globally optimal” regimen for that person.

Here we describe novel analytical and numerical methods to identify person-specific, globally optimal prophylaxis regimens for children with hemophilia A. We present methods to identify prophylaxis regimens that maximize time above a threshold factor VIII concentration, maximize the lowest factor VIII concentration, and minimize the risk of bleeds.

## Methods

### Pharmacokinetics of prophylaxis

The first step in identifying optimal prophylaxis regimens is to write equations that describe the time course of changes in plasma factor VIII concentrations (the ‘pharmacokinetic trajectory’) produced by any prophylaxis regimen. The equations must be able to describe the steady state pharmacokinetic trajectories produced by prophylaxis regimens that involve unequal doses and unequal time intervals between injections.

We make the widely used assumption that factor VIII pharmacokinetics can be described with a single-compartment linear model [7, 8], recognising that this is only an approximation for conventional factor VIII products [9] and does not apply to extended half-life products [10]. The prophylaxis regimen consists of a repeated cycle of intravenous injections. Time, *t*, is measured from the start of the cycle. The duration of the cycle is *T*. Multiple injections, indexed as *i* = 1 … *j*, are administered in each cycle. Injections may be given at unequal intervals and may be of different doses. Injection *i* is given at time *t*_*i*_. In this first section on pharmacokinetic optimization, the cycle is considered to start at the time of one of the injections. Consequently *t*_*1*_, the time of the first injection, *= 0*. Each injection is of dose *D*_*i*_. People with hemophilia A may produce endogenous factor VIII. It is assumed the endogenous factor VIII concentration, *E*, is constant over time.

Consider a person with hemophilia given his first injection of factor VIII. Immediately after the injection, plasma factor VIII concentration, *C*, increases by the product of the dose, *D*_*F*_, and the in vivo recovery, *IVR*. The subscript *F* is used to indicate that that this is the first-ever injection. Subsequently the factor VIII concentration declines exponentially with time constant *τ* (equal to the factor VIII half-life / ln[2]) until a trough concentration (i.e., a local minimum) is reached just before the next injection. Over the period between the first and second injections,

$$C_{F}=E+IVR\;D_{F}\;e^{-t/\tau }$$

With repeated identical prophylaxis cycles the system approaches a steady state in which the pharmacokinetic trajectory is the same on each cycle. For the rest of this paper it is assumed that a steady state has been achieved.

At steady state, the trough concentration that immediately precedes injection *i* is *E + G*_*i*_. The quantity *G*_*i*_ is the residual factor VIII concentration, at the time of injection *i*, ‘left over’ from all preceding injections. S1 Appendix shows that, at steady state in a cycle of *j* injections, the trough preceding any particular injection, *n*, in the prophylaxis cycle is

$${E+G}_{n}=E+\left(\sum_{i=1}^{j}\;{{IVR\;D}_{i}\;e}^{(t_{i}-T-t_{n})/\tau }\right)/\left(1-e^{-T/\tau }\right)$$ (Eq 1)

Each of the *G*_*i*_ can be calculated by subtracting *E* from both sides of this equation. Once the *G*_*i*_ have been calculated, the factor VIII concentration at time *t* in the period between injections *t*_*i*_ and *t*_*i+1*_ can be determined from

$$C=E+(G_{i}+{IVR\;D}_{i})e^{(t_{i}-t)/\tau }$$ (Eq 2)

Here, as elsewhere in this paper, *t*_*i+1*_ is understood to mean *T + t*_*1*_ when *i = j*. Together, Eqs 1 and 2 describe the steady state pharmacokinetic trajectory for any prophylaxis regimen.

### Optimization of pharmacokinetic objectives

If we are to identify optimal prophylaxis regimens we must nominate the criterion by which optimality is to be assessed. We begin by considering ways in which a prophylaxis regimen may be *pharmacokinetically* optimal. We could say that the prophylaxis regimen is optimal if it maximizes the time for which factor VIII concentrations exceed some threshold, *L* (e.g., 1 IU/dL). Alternatively, we could say that the prophylaxis regimen is optimal if it maximizes the lowest factor VIII concentration.

At steady state, the time above threshold in the period between injections *t*_*i*_ and *t*_*i+1*_ is

$$|\begin{array}{lll} t_{Li}=t_{i+1}-t_{i} & \mathrm{if} & E+\left(G_{i}+IVR\;D_{i}\right)\;e^{\left(t_{i}-t_{i+1}\right)/\tau }>L\\ t_{Li}=-\tau \;ln\left[\left(L-E\right)/\left(G_{i}+IVR\;D_{i}\right)\right] & \mathrm{if} & E+\left(G_{i}+IVR\;D_{i}\right)>L\\ & \mathrm{and} & E+\left(G_{i}+IVR\;D_{i}\right)\;e^{\left(t_{i}-t_{i+1}\right)/\tau }\leq L\\ t_{Li}=0 & \mathrm{if} & E+\left(G_{i}+IVR\;D_{i}\right)\leq L \end{array}$$ (Eq 3)

Total time above threshold in each cycle is then

$$t_{L}=\sum_{i=1}^{j}\;t_{Li}$$ (Eq 4)

The lowest factor VIII concentration in a cycle is

$$M=min(E+G_{1},E+G_{2},E+G_{3})$$ (Eq 5)

### Identification of pharmacokinetically optimal prophylaxis regimens

Two approaches were used to identify pharmacokinetically optimal prophylaxis regimens.

The first approach was to write analytical expressions identifying the regimens that maximize the lowest factor VIII concentrations. These equations are presented in S2 Appendix. We were able to derive analytical expressions for the regimens that maximize the lowest factor VIII concentrations but were unable to derive general analytical expressions for the regimens that maximize time above threshold.

The alternative approach–a numerical approach—involved grid searching. To conduct a grid search we first generated a multi-dimensional “grid” consisting of many different prophylaxis regimens, each with a different combination of injection times and doses. Then, for each of those prophylaxis regimens, Eqs 1–5 were used to calculate the time above threshold and the lowest factor VIII concentration in a prophylaxis cycle. The regimens that generated the longest time above threshold or the highest trough levels were considered to be optimal.

One advantage of the grid searching approach is that it explicitly evaluates time above threshold and the lowest factor VIII concentration for the whole range of potential prophylaxis regimens, not just the optimal regimen. That makes it possible to determine the extent to which particular sub-optimal prophylaxis regimens are inferior to the optimal regimen. Contour plots were used to display the effects of prophylaxis regimens on time above threshold or the lowest factor VIII concentration. With these graphs it is possible to explore the trade-off between the efficacy and the practicality or acceptability of particular prophylaxis regimens.

In this paper we report on grid searches conducted to identify the best prophylaxis regimens from amongst the set of prophylaxis regimens in which a total of 90 IU/kg is administered in 1, 2, or 3 injections per week. Except where indicated, all of the calculations reported here are for a person who has no endogenous factor VIII, an in vivo recovery of 2.0 kg/dL and a half-life of 10.7 hours (i.e., *τ* = 15.4 hours) [11]. For calculations of time above threshold, a threshold of 1 IU/dL was used. While we report only on grid searches conducted for this specific set of prophylaxis regimens on a person with this specific pharmacokinetic profile, the approach used here can be applied to prophylaxis regimens that involve any total dose, number of injections, cycle duration or threshold, and it can be applied to people with any endogenous factor VIII concentration, in vivo recovery or half-life.

As all of the prophylaxis regimens reported here involved a weekly prophylaxis cycle, the timing of the injections could be defined by just two parameters: the delay between the first and second injection and the delay between the first and third injection. Likewise, as there were up to three injections per week and the total weekly dose was always 90 IU/kg, the doses could be defined with just two parameters: the doses of the first and second injections. Consequently it was necessary to optimize four parameters. The grid searches evaluated regimens that involved injections with doses of 0, 5, 10, 15, … 90 IU/kg. Injections were given on the half-hour. This resulted in a four-dimensional grid of 10,757,040 prophylaxis regimens.

### Optimization (minimization) of risk of bleeds

Minimization of the risk of bleeds requires an explicit model of the relationship between factor concentration and the risk of bleeds. In this paper we use a model developed in a case-crossover study conducted by Broderick and colleagues [12]. However the general approach used here is not constrained only to the use of Broderick’s model. Later in this paper we consider modification of that model.

Broderick and colleagues studied 104 children with moderate or severe hemophilia A, all of whom were followed for one year. The case-crossover design provided strong control of confounding from within-subject characteristics and some control for confounding by physical activity. A limitation of the study was that pharmacokinetic data were not available for individual children so population-averaged values were used. Broderick’s model related incidence rate of bleeding, *R*, over a short period of time (minutes or hours) for person *q* at time *t* to the product of the person-specific baseline bleed rate, *BBR*_*q*_, and a person- and time-specific incidence rate ratio, *IRR*_*qt*_

$$R_{qt}={BBR}_{q}\;{IRR}_{qt}$$

The baseline bleed rate is the (possibly hypothetical) rate of bleeding the person experiences when not engaged in moderate- or high-risk physical activity and when there is no factor VIII in the blood. This variable provides a proxy for many known and unknown variables that influence the person’s propensity to bleed. The incidence rate ratio is a time-dependent factor by which the risk of bleeding is elevated above or reduced below the baseline rate of bleeding at a particular point in time. Subsequently we focus on clinical outcomes for a particular person so for simplicity we suppress the subscript *q*.

In Broderick’s model the incidence rate ratio for a particular child was assumed to vary over time as a function of two factors: factor VIII concentration and the level of physical activity. Specifically,

$$IRR_{t}=e^{a\;C_{t}}\;e^{b\;Cat2_{t}}\;e^{c\;Cat3_{t}}$$ (Eq 6)

where *Cat2*_*t*_ and *Cat3*_*t*_ are binary variables with values of 1 or 0 indicating whether the child is or is not participating in physical activities thought to increase risk of bleeds at time *t* [12]. Category 2 activities are those in which significant collisions might occur (e.g., basketball) and category 3 activities are those in which significant collisions are inevitable (e.g., skateboard riding). *a*, *b* and *c* are constants that scale the risk of bleeds associated with factor level and physical activity.

S3 Appendix derives an expression (Equation A3.4) that relates the incidence rate ratio to the time course of changes in factor VIII concentration and patterns of physical activity

$$IRR=\sum_{I}\left[-\tau \;e^{b\;{Cat2}_{I}}\;e^{c\;{Cat3}_{I}}\;e^{a\;E}\;(E_{i}\{k\;e^{-(t_{I}^{\prime }-t_{P})/\tau }\}-E_{i}\{k\;e^{-(t_{I}^{\prime \prime }-t_{P})/\tau }\})\right]/T$$

where $E_{i}\{kz^{*}\}$ is the exponential integral $\int_{z^{*}}^{\infty }\frac{e^{kz}}{z}\;dz$, *k* = *a* (*G*_*P*_ + *IVR D_P_*), and the subscript *P* denotes the preceding injection. The subscript *I* indexes each of the intervals in a cycle; a new interval commences when there is an injection or when there is a change in physical activity category. Each interval starts at an instance of the variable *t’* and ends at an instance of the variable *t”*. S3 Appendix also describes a method for calculating the expected annualized bleed rate.

In their case-crossover study, Broderick and colleagues estimated the natural log of parameters *a*, *b* and *c* and reported values of *ln(a)* = 0.980 kg/IU, *ln(b)* = 2.7 and *ln(c)* = 3.7. However that analysis was conducted on case and control windows which consisted of a single epoch of 8 hours duration. Eq 6 and A3.4 require that *a*, *b* and *c* reflect the instantaneous risk. For that reason the original data were re-analysed using 8 hour case and control windows consisting of 15 minute epochs. It was thought that estimates of *a*, *b* and *c* based on 15 minute epochs (the shortest duration epoch for which data were available) would more closely reflect the instantaneous risk. This analysis yielded values of *ln(a)* = 0.985 kg/IU, *ln(b)* = 3.4 and *ln(c)* = 5.6. Those values were used in the primary analyses reported below.

Additional analyses were conducted using a modification of Broderick’s model. The justification for modifying Broderick’s model is that some observational data suggest the fall in risk of bleeds with increases in factor VIII concentration is more rapid than implied by the Broderick model. For example, den Uijl and colleagues [5] collated data on the annual incidence of joint bleeds in 377 patients aged 0–31 years with hemophilia A. The pooled estimate of the annual incidence of joint bleeds was around 6, but the incidence was much lower in people with endogenous factor VIII concentrations greater than 12 IU/dL. The relationship between endogenous factor VIII concentration and incidence of joint bleeds observed by den Uijl and colleagues is likely to be confounded by prophylaxis. Nonetheless the data suggest that, in contrast to the estimated risk function for all bleeds obtained by Broderick and colleagues, the risk of joint bleeding is nearly eliminated once factor VIII concentrations exceed ~10 IU/dL. Our calculations also suggest the effect of factor VIII on risk of bleeds implied by the observational data analysed by Collins and colleagues [4] and the effects of prophylaxis on bleeds incidence reported in randomized trials [1, 2] is larger than the effect implied by Broderick’s model. Consequently additional calculations were conducted using a modification of Broderick’s model that made the risk of bleeds fall more rapidly with increasing factor VIII concentrations. Instead of using the value of 0.985 for the parameter ln(*a*), a value of 0.870 was used. This value was selected because it implies risk decays by 75% with a factor VIII concentration of 10 IU/dL which is roughly consistent with the data presented by den Uijl and colleagues (see their Figure 1).

### Identification of the prophylaxis regimen that minimizes risk of bleeds

Grid searches were conducted to identify prophylaxis regimens that minimize the incidence of bleeds. The grid search involved using Equation A3.4 to evaluate the risk of bleeds for each of a large number of prophylaxis regimens. The prophylaxis regimen associated with the lowest risk of bleeds was considered optimal.

To generate the data reported here, it was assumed that the prophylaxis cycle involved up to three injections in a one-week cycle (*T* = 168 hours) administered to a person whose factor VIII half-life was 10.7 hours and who had an in vivo recovery of 2.0 kg/dL and no endogenous factor VIII. These values for half-life and in vivo recovery are similar to the mean values reported by Barnes and colleagues [11]. While we report only on grid searches conducted for this specific set of prophylaxis regimens on a person with this specific pharmacokinetic profile, the approach used here can be applied to other prophylaxis regimens and to people with other pharmacokinetic profiles.

For a prophylaxis cycle consisting of up to three injections it was necessary to optimize five parameters: the time of week of the first, second and third injection and the doses of the first and second injections. To prevent the grid on which searches were conducted from becoming unmanageably large, a relatively coarse grid was used. The grid consisted of doses of 0, 15, 30, 45, 60, 75 or 90 IU/kg per injection with the constraint that the three doses must total 90 IU/kg. Injections were given on the hour. Additional constraints were that injections could not occur while the person was asleep (in the calculations reported here, the person was assumed to sleep from 10:00 pm to 5:00 am) or while the person was participating in category 2 or category 3 physical activity. The size of the grid depended on how active the person was (see below) but most grids consisted of ~1–2 million prophylaxis regimens. Grid searches were conducted with Matlab R2014b. The main findings were replicated in Stata 14.1.

To illustrate the consequences of different physical activity patterns, separate grid searches were conducted for hypothetical children with different patterns of physical activity: one was a ‘very active’ child who participated in category 2 activity every weekday from 4:00–6:00 pm and on weekends from 10:00–12:00 and category 3 activities on Tuesdays and Thursdays from 6:00–7:00 pm and on Saturdays and Sundays from 3:00–5:00 pm; the second was an ‘inactive’ child who participated in category 2 activity on Tuesdays and Thursdays from 5:00–6:00 pm, and the third was a ‘weekend active’ child who participated in category 2 activity on Saturdays and Sundays from 10:00 am-12:00 pm and in category 3 activities on Saturdays and Sundays from 3:00–5:00 pm. These hypothetical patterns of physical activity are not intended to be representative of classes of people. They are simply used as examples to illustrate how the pattern of physical activity might influence the optimal prophylaxis regimen.

A summary of the nomenclature used in this paper is provided in Table 1.

**Table 1:** Table of symbols.

*a*	A coefficient that determines the haemostatic efficacy of exogenous factor VIII.
*b*	A coefficient that determines how much category 2 physical activity changes bleed risk.
*BBR*	The baseline bleed rate.
*c*	A coefficient that determines how much category 3 physical activity changes bleed risk.
*C*_*t*_	Plasma concentration of factor VIII concentration at time *t*.
*D*	The total dose of exogenous factor VIII administered in a prophylaxis cycle.
*D*_*i*_	The dose of the *i*th injection.
*E*	Plasma concentration of endogenous factor VIII concentration.
E	The exponential integral.
*G*_*i*_	Residual plasma concentration of factor VIII at the time of the *i*th injection.
*IRR*_*t*_	The incidence rate ratio at time *t*.
*IVR*	In-vivo recovery.
*j*	The number of injections in a prophylaxis cycle.
*L*	Threshold plasma concentration of factor VIII.
*λ*	The Lagrangian multiplier.
*M*	The lowest plasma concentration of factor VIII in a prophylaxis cycle.
*R*	The incidence rate of bleeding.
*t*	Time.
*t*_*i*_	Time of the *i*th injection.
*t*_*L*_	Time spent above threshold in a prophylaxis cycle.
*t*_*Li*_	Time spent above threshold since the injection preceding injection *i*.
*t*_*j*_	The time at which a prophylaxis cycle ends.
*t'*	The time at which an interval starts.
*t''*	The time at which an interval ends.
*T*	Duration of a prophylaxis cycle.
*τ*	Time constant = half-life / ln(2).

## Results

### Maximizing time above threshold

Grid searching indicated that the time above a threshold of 1 IU/dL was maximized by giving injections of equal doses at equal intervals. Thus prophylaxis was optimal, in the sense of maximising time above threshold, when the second and third injections were given 56 and 112 hours after the first. With a weekly dose of 90 IU/kg, this prophylaxis regimen kept factor VIII concentrations above 1 IU/dL for all 168 hours per week.

**Fig 1:**
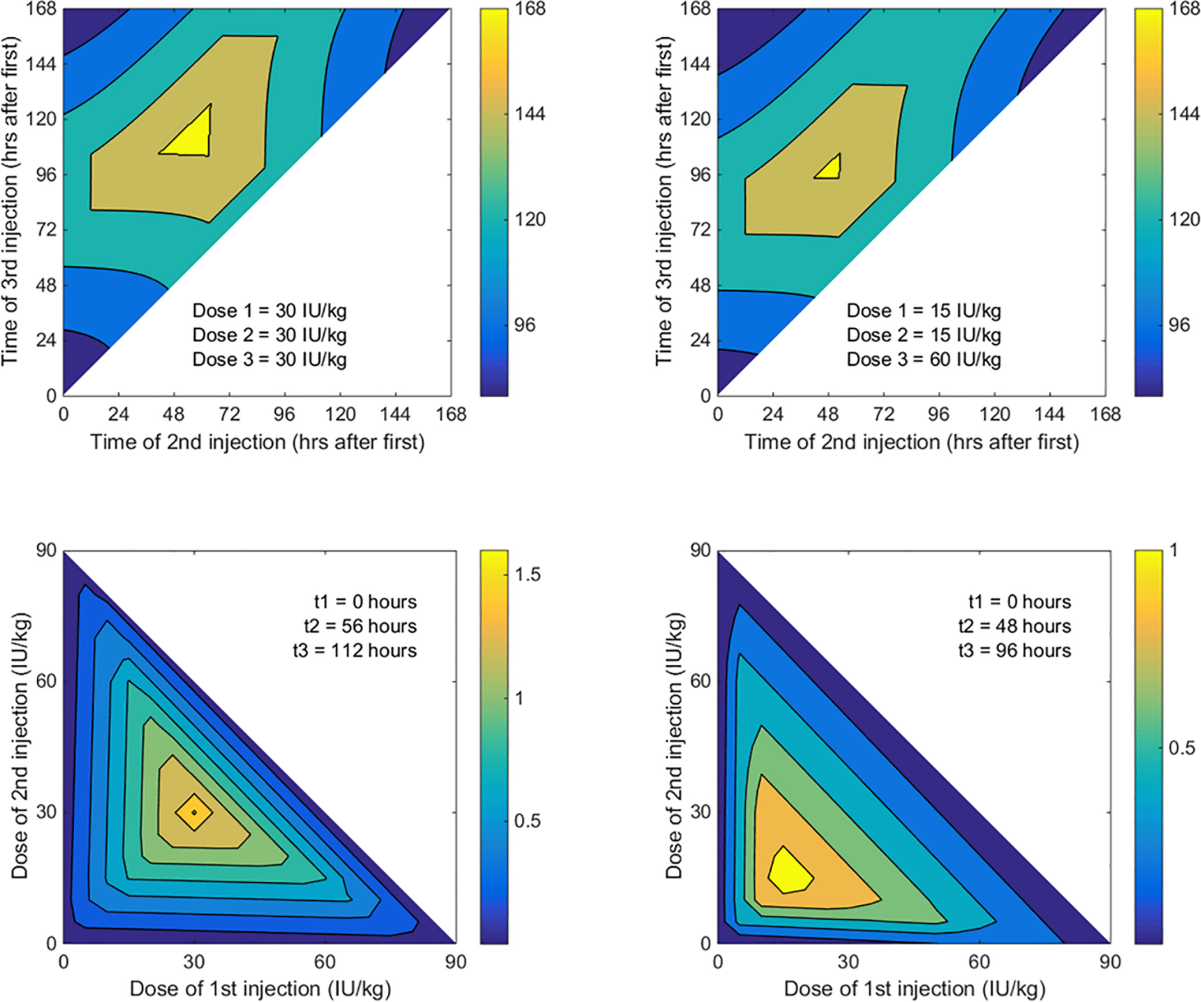
Effect of timing and dose of injections on time (in hours per week) above a threshold of 1 IU/dL. Panels A and B show the effect of timing of injections when the dose of injections is fixed. Panels C and D show the effect of dose of injections when the timing of injections is fixed. In panels A and C, the dose of all three injections is equal (*D*_*1*_ = *D*_*2*_ = *D*_*3*_ = 30 IU/kg). In panels B and D, the dose of injections is unequal (*D*_*1*_ = *D*_*2*_ = 15 IU/kg; *D*_*3*_ = 60 IU/kg). The threshold is 1 IU/dL. In this and subsequent figures, three injections of a total of 90 IU/kg are given in a weekly cycle to a person with *E =* 0 IU/dL, IVR = 2 kg/dL, and a half-life of 10.7 hours.

Other regimens can also keep factor VIII concentrations above threshold for 168 hours per week, so they too are optimal. This is illustrated in Fig 1A which shows the effect of the timing of injections on time above threshold when all three injections are the same dose. When the three doses are equal, all of the combinations of timing of injections that fall within the yellow triangle in the centre of the figure keep factor VIII concentrations continuously above threshold.

Fig 1A also shows the consequences of administering injections at unequal intervals while keeping the doses equal. For example, if the second and third injections are administered 48 and 96 hours after the first injection (i.e., at the same time of day, two and four days after the first injection), time spent above 1 IU/dL concentration is 160 hours per week. That prophylaxis regimen is suboptimal, at least as defined here, because it causes the factor VIII concentrations to drop below 1 IU/dL for 8 of the 168 hours in a week.

Fig 1B shows the same scenario as Fig 1A except that now the total dose of 90 IU/kg/week is shared across the three injections in unequal doses: doses of the first, second and third injections are 15, 15 and 60 IU/kg/week, in that order. Comparison of Fig 1A and 1B demonstrates two interesting findings. First, it is possible to administer prophylaxis optimally using unequal doses, at least if optimality is defined in terms of time above threshold. However, the domain of optimal combinations of timings of injections is more restricted with unequal dose injections (the yellow triangle in Fig 1B is smaller than that in Fig 1A). A second finding is that the optimal timing with these doses is not very different from the optimal timing with equal doses. That is, the optimal timing of doses appears to be insensitive to how the total dose is shared across injections. Nonetheless, in contrast to when injections of equal dose are given, factor VIII concentrations can be kept continuously above 1 IU/dL with unequal dose injections by giving the second and third injections at the same time of day (i.e., 48 and 96 hours after the first). Also, in contrast to when equal doses are given, it is not optimal with these unequal doses to give injections at equally spaced intervals: if the second and third injections are given 56 and 112 hours after the first, factor VIII concentrations drop below 1 IU/dL for 5 hours per week.

Fig 1C shows the effect of varying dose across injections if the injections are given at 56 and 112 hours (i.e., at equal intervals). With this injection schedule, time above threshold is insensitive to how the dose is shared between injections. If, however, the second and third injections are given at the same time of day (48 and 96 hours after the first; a schedule that is likely to be much more acceptable than the equal interval schedule) the domain of optimal combinations of doses of injections is much smaller. Fig 1D shows that if the injections are to be given at these times the doses must be close to 15, 15 and 60 IU/kg.

### Maximizing the lowest factor VIII concentration

S2 Appendix demonstrates analytically that, given a fixed total dose and a fixed injection schedule, there is always a single regimen that maximizes the lowest factor VIII concentration. That regimen involves sharing doses between injections in a way that ensures all of the troughs are equal. It is also shown that, of all possible injection schedules, the schedule which maximizes the lowest factor VIII concentration is one in which injections are given at equal intervals. Thus the regimen which maximizes the lowest factor VIII concentration is one in which equal doses are administered at equal intervals. For a given total dose, the lowest factor VIII concentration increases with increasing numbers of injections. With an infinite number of injections at equal intervals (i.e., with a continuous infusion) the lowest factor VIII concentration is *IVR D τ / T*.

**Fig 2:**
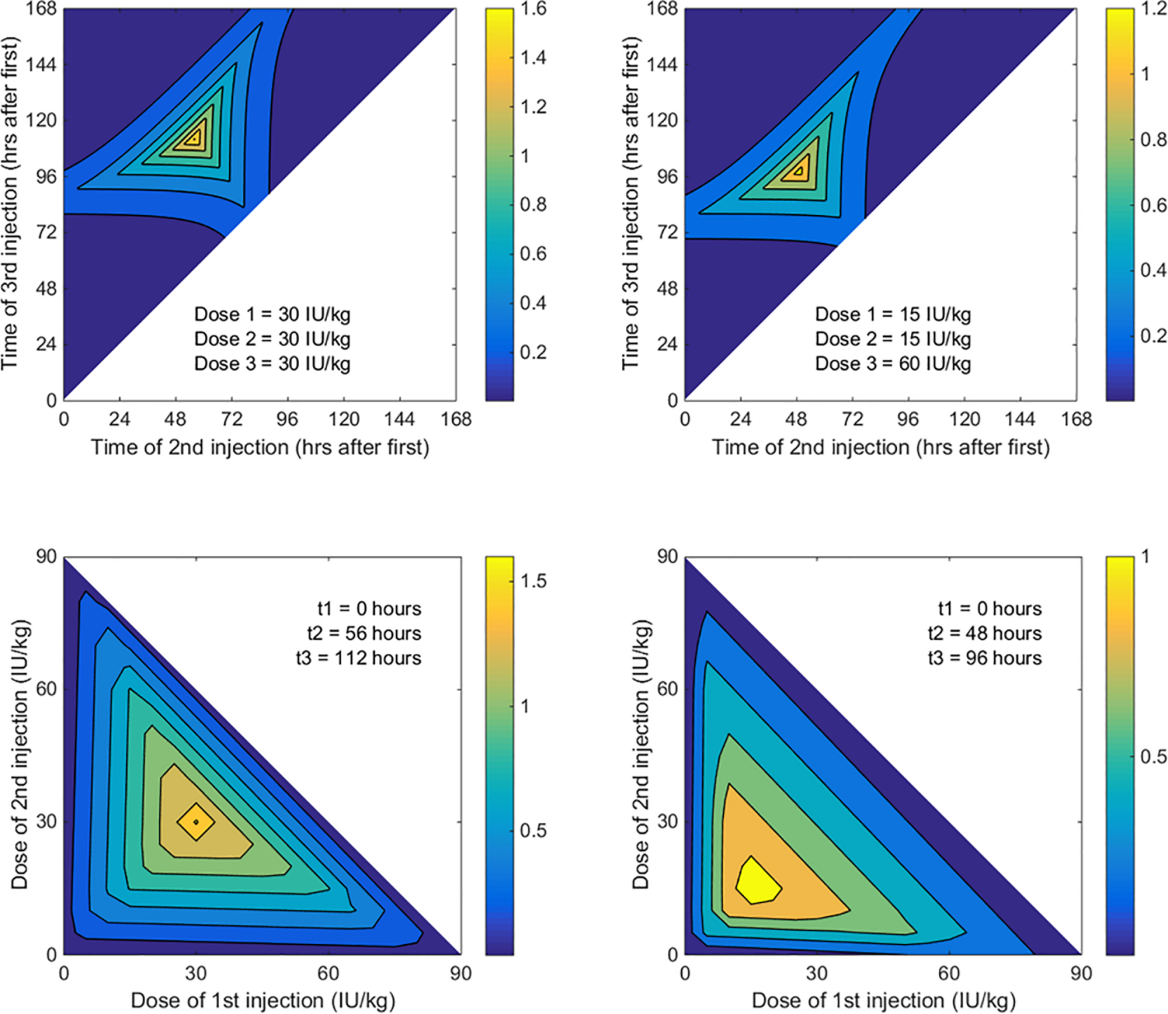
Effect of timing and dose of injections on the lowest factor VIII concentration. Panels A and B show the effect of timing of injections when the dose of injections is fixed. Panels C and D show the effect of dose of injections when the timing of injections is fixed. In panels A and C, the dose of all three injections is equal (*D*_*1*_ = *D*_*2*_ = *D*_*3*_ = 30 IU/kg). In panels B and D, the dose of injections is unequal (*D*_*1*_ = *D*_*2*_ = 15 IU/kg; *D*_*3*_ = 30 IU/kg).

The grid search provides further information about how the prophylaxis regimen influences the lowest factor VIII concentration. Fig 2A shows that when three equal doses are administered at equal intervals the lowest factor VIII concentration is 1.6 IU/dL. If, instead, the second and third injections are given at the same time of day (48 and 96 hours after the first), the lowest factor VIII concentration is reduced to 0.6 IU/dL.

S2 Appendix shows that if the doses are unequal then it is no longer optimal, in the sense of maximizing the lowest factor VIII concentration, to give injections equally spaced in time; the timing is optimized when the troughs are equal. Fig 2B shows that when doses of 15, 15 and 60 IU/kg are administered, the second and third injections are best administered 49 and 98 hours after the first (i.e., at nearly the same time of day on the second and fourth day after the first injection). This regimen causes all trough concentrations to equal 1.3 IU/dL.

When the three injections are of equal dose, the timing of injections which maximizes the lowest factor VIII concentration is independent of the half-life. However, when the injections are of unequal doses the optimal timing varies slightly with half-life. For example, with doses of 15, 15 and 60 IU/kg, the optimal timing of the second and third injections is 49 and 98 hours after the first injection for a person with a half-life of 10.7 hours, but the optimal timing of the second and third injections is 50 and 101 hours after the first injection for a person with a half-life of 8 hours, and 47 and 94 hours after the first injection for a person with a half-life of 15 hours.

### Minimising risk of bleeds

**Fig 3:**
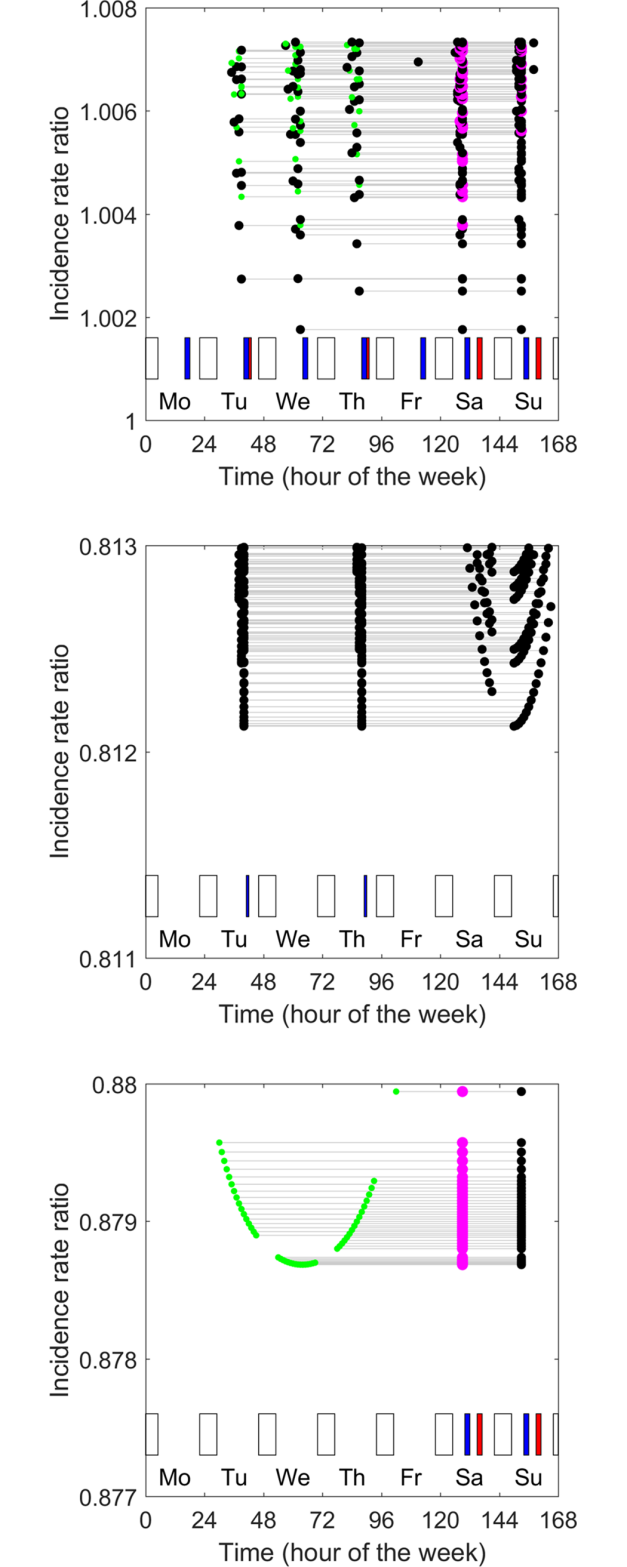
Optimal prophylaxis regimens identified using Broderick’s model. A, a ‘very active’ child, B, an ‘inactive’ child, and C, a ‘weekend active’ child. In each panel, the 100 prophylaxis regimens that best minimize bleeds incidence are shown. Each prophylaxis regimen is shown by a horizontal line joining three circles. The horizontal location of the symbols indicates the timing of the injections for each prophylaxis regimen and the vertical location indicates the incidence rate ratio associated with the prophylaxis regimen (lower values indicate lower incidence of bleeds). Time is expressed as hour of the week, starting at midnight on Sunday night. Patterns of physical activity are shown as bars in the lower part of each panel. Blue bars are periods of category 2 activity. Red bars are periods of category 3 activity. Unfilled bars are periods of sleep. The size and colour of the circles indicates the dose (small green circles 15 IU/kg, intermediate black circles 30 IU/kg, large pink circles 45 IU/kg).

Fig 3A shows the 100 best prophylaxis regimens (from amongst 1,040,239 prophylaxis regimens) for the ‘very active’ child. Here ‘best’ refers to the prophylaxis regimens that confer the lowest incidence of bleeds. There is very little difference in the incidence rate of bleeds conferred by the best 100 prophylaxis regimens: the incidence rate ratios of these 100 regimens varied by less than 1% (from 1.002 to 1.008). In fact the very worst of all prophylaxis regimens (a single injection of 90 IU/kg administered on Sunday at 6:00 pm) conferred only a slightly higher incidence of bleeds (*IRR* = 1.15). The implication is that, for this child, any regimen with a total dose of 90 IU/kg administered in up to three injections per week confers a similar risk of bleeds.

The optimal prophylaxis regimen for this ‘very active’ child involves injections of 30, 30 and 30 IU/kg administered on Wednesday at 3:00 pm, Saturday at 9:00 am and Sunday at 9:00 am. The *IRR* of 1.002 implies that this regimen keeps the incidence of bleeds very close to the incidence the child would have experienced if he did not participate in category 2 or category 3 activity and had no factor in his blood, even though he was very active.

In all of the best 100 prophylaxis regimens, injections were given in doses of between 15 and 45 IU/kg. In just under half (46/100) of these regimens the doses were unequal. None involved doses of 0 (i.e., none involved just one or two injections). All of the best 100 prophylaxis regimens involved injections on Saturday morning before participating in category 2 and 3 activity, and all involved injections on Sunday, usually but not always just before participating in category 2 and 3 activity. Also, all of the best 100 prophylaxis regimens involved giving one injection on a week day, almost always in the afternoon soon before participating in category 2 or category 3 physical activities.

Comparison of the three panels in Fig 3 shows that the optimal prophylaxis regimens for the ‘very active’ child (Fig 3A), the ‘inactive’ child (Fig 3B) and the ‘weekend active’ child (Fig 3C) are quite different. For example, the optimal prophylaxis regimens for the inactive child involve the administration of equal doses on Tuesday at 4:00 pm, Thursday at 4:00 pm and Sunday at 6:00 am. The optimal prophylaxis regimen for the ‘weekend active’ child involves an injection of 15 IU/kg on Wednesday afternoon at 4:00 pm, an injection of 45 IU/kg on Saturday at 9:00 am and an injection of 30 IU/kg on Sunday at 9:00 am. Thus the optimal prophylaxis regimen is sensitive to patterns of physical activity.

Many of the optimal and near-optimal prophylaxis regimens are quite different to the regimens that are pharmacokinetically optimal, and quite different from regimens that are typically prescribed. For example, optimal prophylaxis for the ‘weekend active’ child considered here involves administering factor VIII in unequal doses, twice on a weekend, and once during the week in the afternoon.

**Fig 4:**
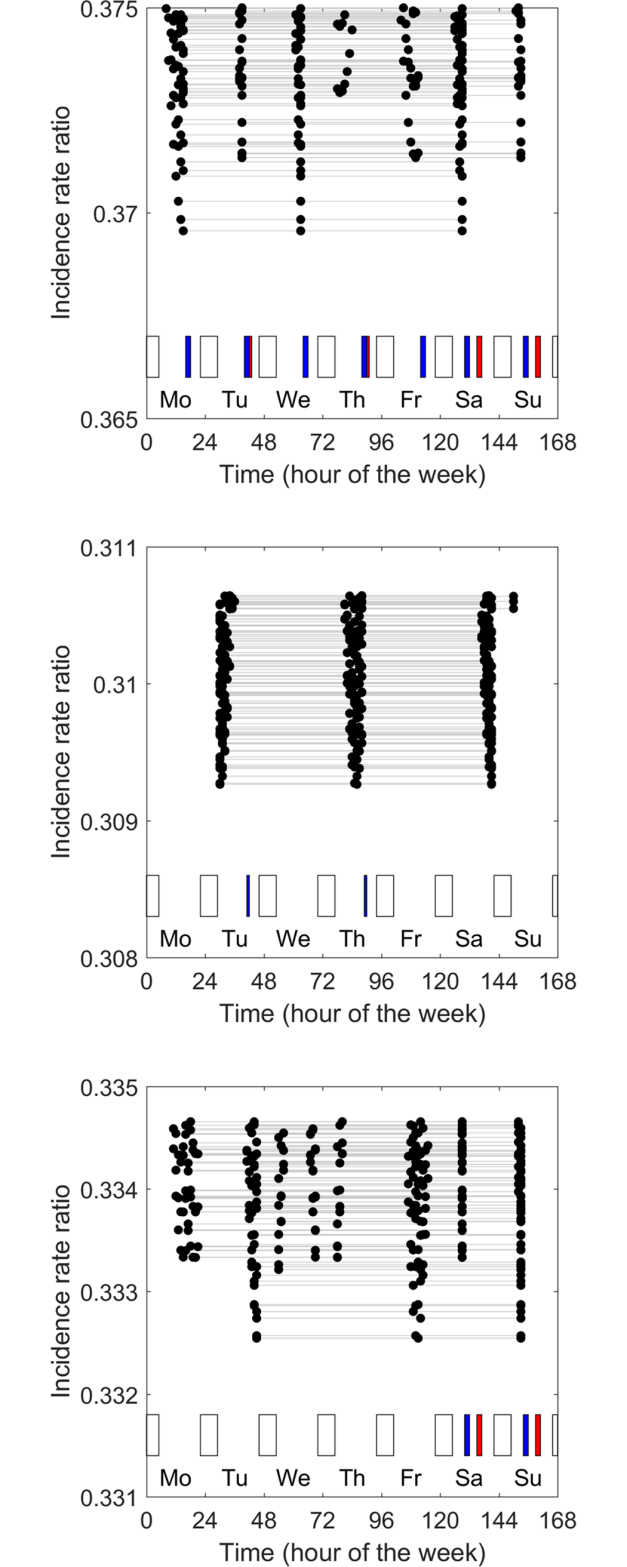
Optimal prophylaxis regimens identified using the modified Broderick’s model. A, a ‘very active’ child, B, an ‘inactive’ child, and C, a ‘weekend active’ child. The figure is the same as Fig 3 except that the effect of factor VIII concentration has been increased (*ln(a) =* 0.870).

Fig 4 shows the 100 best prophylaxis regimens identified when Broderick’s model was modified by increasing the effect of factor VIII concentration on risk of bleeds. The effect is not only to lower the risk of bleeds (compare the y axis values in 4A-C with those in 3A-C) but also to change the ordering of the efficacy of the prophylaxis regimens. A general observation is that, when the effect of factor VIII concentration on risk of bleeds is increased, both the optimal doses and optimal spacing of injections become more uniform, and the optimal timing of injections becomes less influenced by the timing of periods of physical activity (compare, for example, Fig 4C with Fig 3C).

**Fig 5:**
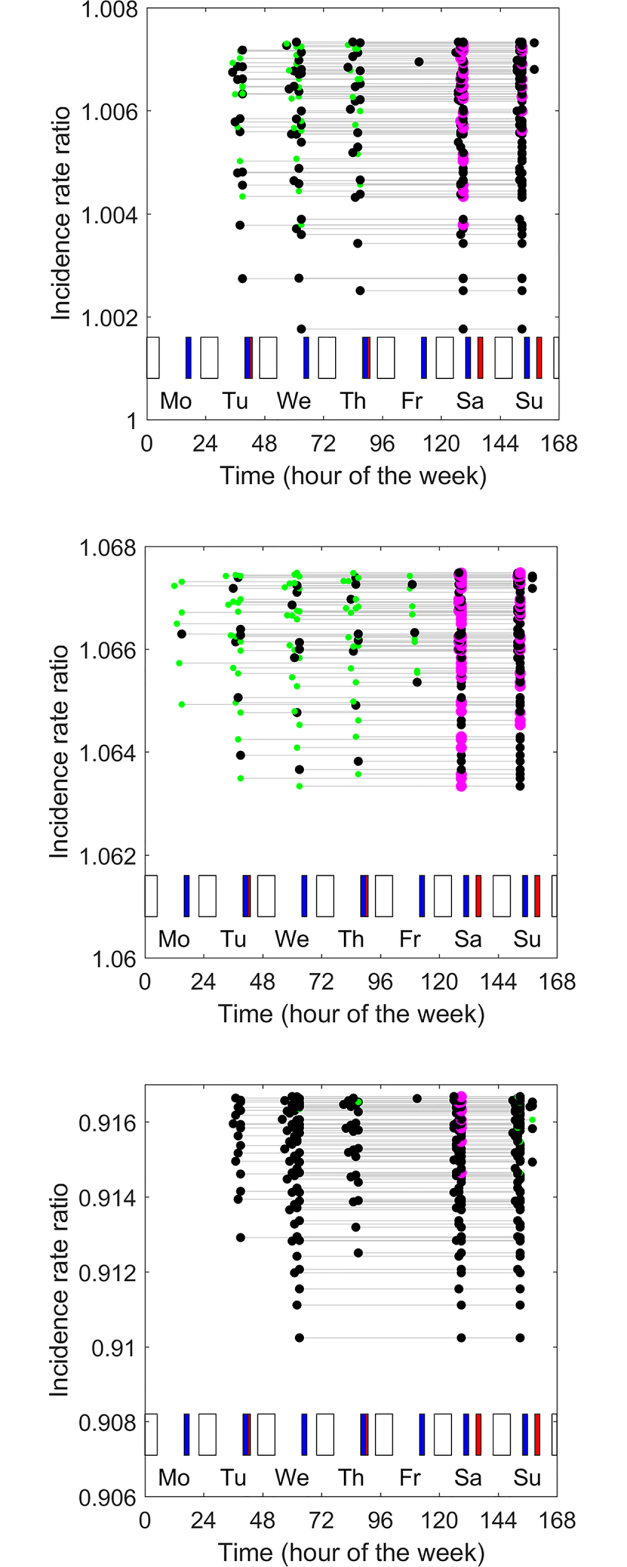
Effect of factor VIII half-life on optimal prophylaxis regimens identified using Broderick’s model for the ‘very active’ child. A, half-life = 10.7 hours (This panel is the same as Fig 3A), B, half-life = 8 hours, and C, half-life = 15 hours.

Fig 5 shows how the prophylaxis regimens that minimize the risk of bleeds may differ across children with different factor VIII half-lives. Using Broderick’s model to identify the optimal prophylaxis schedule for a ‘very active’ child, it is shown that there are some differences, but not large differences, between the optimal prophylaxis schedules for children with half-lives of 8, 10.7 and 15 hours. Interestingly, the optimal timing of the doses is similar, but total dose must be shared more equally across injections for children with longer factor VIII half-lives. Comparison of Fig 5 also demonstrates that optimal prophylaxis has a greater effect on relative risk of bleeds (relative, that is, to baseline bleed rate) in children with long Factor VIII half-lives.

## Discussion

This paper describes analytical and numerical methods for identifying optimal person-specific prophylaxis regimens for people with hemophilia. New equations were written for (a) the steady state pharmacokinetic trajectory of factor VIII for a prophylaxis regimen in which the doses of injections and intervals between injections may be unequal; (b) time above threshold, the lowest factor VIII concentrations and risk of bleeds for any prophylaxis regimen; and (c) the prophylaxis regimen that maximizes the lowest factor VIII concentration.

### Limitations

There are several limitations of the current work. The first is that the equations used here assume the pharmacokinetics of factor VIII injections can be represented by a single compartment linear model. This assumption has been used extensively in studies of hemophilia prophylaxis. However population pharmacokinetic models have demonstrated that the pharmacokinetics of recombinant factor VIII are more accurately represented by a two-compartment model [9]. Extended half-life products also have more complex pharmacokinetics [10, 13]. We are currently working on extending the methods used here to more complex pharmacokinetic models.

Grid searching was used to identify optimal prophylaxis regimens. A limitation of grid searching is that if the grid is not fine enough the optimal prophylaxis regimen could fall between points on the grid, in which case the grid search could fail to identify the true optimum. Another limitation is that grid searching can be slow and computationally intensive. As an alternative to grid searching, we considered using either constrained non-linear optimization (the fmincon function in Matlab R2014b) or a sophisticated global search strategy (the GlobalSearch function in Matlab R2014b) to identify pharmacokinetically optimal prophylaxis regimens. However, neither the fmincon nor the GlobalSearch function performed well in this context: both consistently identified prophylaxis regimens whose factor VIII concentrations were slightly lower than the lowest concentrations associated with the optimal prophylaxis regimens identified with grid searches. This is probably because the objective functions described in Equations 3–5 are non-smooth (i.e., not twice differentiable).

The methods used to identify prophylaxis regimens that minimize risk of bleeds have some additional limitations. One is that the optimal prophylaxis regimen is sensitive to the estimate of the effect of factor VIII concentration on risk of bleeds. The estimate was obtained from a case-crossover study which provided strong protection from confounding by time-invariant variables. Nonetheless the estimate could still be biased by residual confounding caused by error in recall of the time of bleeds, the time of injections, or the timing and intensity of physical activity. Another potential source of bias was the estimation of individuals’ factor VIII concentrations using population average pharmacokinetic parameters. Also, the relative effect (but not the absolute effect) of factor VIII concentration on risk of bleeds was assumed to be the same across all subjects [12]. While these considerations suggest that the specific predictions about optimal prophylaxis regimens reported here should be interpreted cautiously, they do not invalidate the general approach. The general approach presented here is not restricted to the use of Broderick’s estimate. It would be possible, with varying degrees of difficulty, to adopt the approach used here to incorporate any relationship between factor VIII concentration and risk of bleeds, including person-specific estimates should those become available.

Broderick’s estimate was derived from a study of children aged 4–18 years. It may be advisable not to use this estimate to make predictions about optimal prophylaxis in adults.

### Insights into prophylaxis

The analytical approach identified several principles that can guide pharmacokinetic optimization of prophylaxis. For a given total dose, the pharmacokinetically optimal prophylaxis regimen is one in which equal doses are given at equal intervals. And, at least when equal doses are given at equal intervals, the lowest factor VIII concentration is maximized by sharing the total dose across as many injections per cycle as possible.

A less obvious finding is that, if injections are to be administered at unequal intervals, the lowest factor VIII concentration is maximized, for a given total dose, when the total dose is shared between injections in a way that make all trough concentrations equal. This means that the largest dose should be provided before the longest interval. Fig 1C, Fig 1D, Fig 2C and Fig 2D show that the optimal dose for a specific injection is related to the duration of the subsequent interval in a complex way. The optimal dose for any one injection depends on the intervals between and doses of all other injections, not just the subsequent injection.

Prophylaxis regimens must be compatible with lifestyles. Usually children will not be able to have injections while at school, and usually adults will not be able to have injections while at work. Neither children nor adults can have injections while they are engaging in physical activity or sleeping. Many people like to establish a routine in which injections are always given at the same time of day. The numerical approach to optimization described in this paper can be used to determine which of a set of *practical* and *acceptable* prophylaxis regimens is optimal.

If injections are to be given at the same time of day (which, in a weekly cycle of three injections, implies that injections will be at unequal intervals), different doses must be given on each day if the lowest factor VIII concentration is to be maximized. For a person with a half-life of 10.7 hours, injections given on Monday, Wednesday and Friday mornings should be given approximately in the ratio 15:15:60. If the person has no endogenous factor VIII and an in vivo recovery of 2 kg/dL, a total dose of 90 IU/kg/week using this regimen will keep the lowest factor VIII concentration above 1.1 IU/dl. Alternatively, the lowest factor VIII concentration could be kept above 1 IU/dl with injections 30, 30 and 30 IU/kg on Monday and Wednesday mornings and Friday afternoons. Equal dose injections given on Monday, Wednesday and Friday mornings will not keep factor VIII concentrations above 1 IU/dL. Equal doses administered at equal intervals would increase the lowest factor VIII concentration to 1.6 IU/dl, but such a schedule would usually be impractical: a schedule in which injections are administered at equal intervals is only possible if the person sleeps for less than 8 hours and is prepared to administer injections just after waking and just before sleeping.

All of the methods presented here identify prophylaxis regimens that are person-specific. With simpler approaches to optimization that maximize pharmacokinetic parameters, person-specificity in optimal prophylaxis regimens arises from person-specific pharmacokinetics (endogenous factor VIII levels, half-life and in vivo recovery). Person-specific optimization of pharmacokinetic parameters is possible when the individual’s pharmacokinetics are known. When minimising risk of bleeds, additional person-specificity arises because the optimal prophylaxis regimen depends on the person’s specific physical activity patterns. It may be possible to account for person-specific physical activity patterns if the person has a regular pattern of physical activity. In young children, whose physical activity patterns may be highly irregular, that may be impossible.

Perhaps the most important finding from this study is that the prophylaxis regimens that minimize risk of bleeds may be very different to prophylaxis regimens that maximize pharmacokinetic parameters (such as time above a threshold factor VIII concentration, or the lowest factor VIII concentration).

Some general observations arise from the simulations conducted using the Broderick model. First, when people are exposed to category 2 and 3 activities, the optimal regimen tends to involve injections given just prior to periods of activity. If physical activity occurs late in the day, the optimal prophylaxis schedule may involve injection in the afternoon. This is at odds with the common practice of administering prophylactic injections in the morning. Also, optimal prophylaxis may involve administering injections in unequal doses, or administering two injections on a weekend.

Our preliminary experience is that there are often many prophylaxis regimens that are very close to optimal. When that occurs it would be reasonable to select the most convenient regimen from amongst those that are near-optimal.

The authors are developing a web-based calculator, called MOrPH, which will conduct the calculations described here. When completed, the calculator will be made freely available to hemophilia physicians. Hemophilia physicians who would like to access the web site should contact the corresponding author. The Matlab code used in the analyses has been made available as Supporting Information (S1 Code).

## Supporting information

S1 Appendix Residual plasma factor VIII concentration. (DOCX)

S2 Appendix Prophylaxis regimens that maximize the lowest factor VIII concentration. (DOCX)

S3 Appendix Estimation of bleeds risk. (DOCX)

S1 Code Matlab code. The zip file contains 6 Matlab scripts which were used to conduct the analyses and generate all of the results reported in this paper. A text file, ReadMe.txt, provides a brief description.
(ZIP)
