# Correction: Optimization of prophylaxis for hemophilia A

**DOI:** 10.1371/journal.pone.0196695

**Published:** 2018-04-30

**Authors:** Robert D. Herbert, Carolyn R. Broderick, Chris Barnes, Laurent Billot, Albert Zhou, Jane Latimer

S1 Appendix, S2 Appendix, and S3 Appendix are corrected for improved readability. Please see the correct S1–S3 Appendices here.

## Supporting information

S1 Appendix Residual plasma factor VIII concentration. (PDF)

S2 Appendix Prophylaxis regimens that maximize the lowest factor VIII concentration. (PDF)

S3 Appendix Estimation of bleeds risk. (PDF)
