# Supplementary material for: Optimization of prophylaxis for hemophilia A
Source: PLoS One. 2018 Feb 15;13(2):e0192783. doi: 10.1371/journal.pone.0192783 (PMC5813962; doi:10.1371/journal.pone.0192783)
Supplement: S3 Appendix — (DOCX) [file pone.0192783.s003.docx]

# $C_{F}=E+IVR D_{F} e^{{-t}/\tau}$ ${E+G}_{n}= E+ \left( \sum_{i=1}^{j} {{IVR D}_{i} e}^{\left( t_{i}-T-t_{n} \right)/\tau} \right)/\left( 1- e^{-T/\tau} \right)$ $C=E+(G_{i}+{IVR D}_{i})e^{\left( t_{i}-t \right)/\tau}$ $t_{Li}={t_{i+1}- t}_{i}$ $t_{Li}=-\tau ln\left[ \left( L-E \right)/\left( G_{i}+{IVR D}_{i} \right) \right]$ $E+\left( G_{i}+{IVR D}_{i} \right) >L$ $t_{Li}=0$ $E+ \left( G_{i}+{IVR D}_{i} \right)\leq L$ $t_{L}=\sum_{i=1}^{j} t_{Li}$ $M=min\left( E+G_{1}, E+ G_{2}, E+ G_{3} \right)$

$$R_{qt}={BBR}_{q} {IRR}_{qt}$$

${IRR}_{t}= e^{a C_{t}} e^{b {Cat2}_{t}} e^{c {Cat3}_{t}}$ $IRR= \sum_{I} \left[ -\tau e^{b {Cat2}_{I}} e^{c {Cat3}_{I}} e^{a E} \left( E_{i}\left\{ k e^{-(t_{I}^{'}-t_{P})/\tau} \right\}- E_{i}\left\{ k e^{-(t_{I}^{''}-t_{P})/\tau} \right\} \right) \right] / T$

$$E_{i}\left\{ kz^{*} \right\}$$

$\int_{z^{*}}^{\infty} \frac{e^{kz}}{z} dz$ $k= a \left( G_{P}+{IVR D}_{P} \right)$ ${E+ G}_{1}= E+\left( G_{1}+ {IVR D}_{1} \right)e^{-T/\tau}$ ${E+ G}_{1}= E+\left[ \left( G_{1}+ {IVR D}_{1} \right)e^{\left( t_{1}-t_{2} \right)/\tau}+{IVR D}_{2} \right]e^{\left( t_{2}-T-t_{1} \right)/\tau}$ ${E+G}_{1}= E+\left\{ \left[ \left( G_{1}+ {IVR D}_{1} \right)e^{\left( t_{1}-t_{2} \right)/\tau}+{IVR D}_{2} \right]e^{\left( t_{2}-t_{3} \right)/\tau}+{IVR D}_{3} \right\}e^{\left( t_{3}-T-t_{1} \right)/\tau}$ ${E+ G}_{1}= E+ \left( G_{1}e^{\left( t_{1}-t_{2} \right)/\tau}e^{\left( t_{2}-t_{3} \right)/\tau}+{IVR D}_{1}e^{\left( t_{1}-t_{2} \right)/\tau}e^{\left( t_{2}-t_{3} \right)/\tau}+ {IVR D}_{2}e^{\left( t_{2}-t_{3} \right)/\tau}+{IVR D}_{3} \right)e^{\left( t_{3}-T-t_{1} \right)/\tau}$

$${E+ G}_{1}= E+G_{1}e^{\left( t_{1}-t_{2} \right)/\tau}e^{\left( t_{2}-t_{3} \right)/\tau}e^{\left( t_{3}-T-t_{1} \right)/\tau}+{IVR D}_{1}e^{\left( t_{1}-t_{2} \right)/\tau}e^{\left( t_{2}-t_{3} \right)/\tau}e^{\left( t_{3}-T-t_{1} \right)/\tau}+ {IVR D}_{2}e^{\left( t_{2}-t_{3} \right)/\tau}e^{\left( t_{3}-T-t_{1} \right)/\tau}+ {IVR D}_{3}e^{\left( t_{3}-T-t_{1} \right)/\tau}$$

$${E+ G}_{1}= E+ G_{1}e^{\left( -T \right)/\tau}+ {IVR D}_{1}e^{\left( -T \right)/\tau}+ IVR D_{2}e^{\left( t_{2}-T-t_{1} \right)/\tau}+ IVR D_{3}e^{\left( t_{3}-T-t_{1} \right)/\tau}$$

$${E+G}_{1}({1-e}^{\left( -T \right)/\tau})=E + IVR {D_{1} e}^{\left( -T \right)/\tau}+ IVR D_{2}e^{\left( t_{2}-T-t_{1} \right)/\tau}+ {IVR D}_{3}e^{\left( t_{3}-T-t_{1} \right)/\tau}$$

$${E+ G}_{1}= {E+\left( {{IVR D}_{1} e}^{\left( -T \right)/\tau}+ {IVR D}_{2}e^{\left( t_{2}-T-t_{1} \right)/\tau}+ {IVR D}_{3}e^{\left( t_{3}-T-t_{1} \right)/\tau} \right)}/\left( 1- e^{\left( -T \right)/\tau} \right)$$

${E+G}_{1}= E+ \left( \sum_{i=1}^{j} {{IVR D}_{i} e}^{\left( t_{i}-T-t_{1} \right)/\tau} \right)/\left( 1- e^{-T/\tau} \right)$ ${E+G}_{n}= E+ \left( \sum_{i=1}^{j} {{IVR D}_{i} e}^{\left( t_{i}-T-t_{n} \right)/\tau} \right)/\left( 1- e^{-T/\tau} \right)$ $G_{i+1}=(G_{i}+{IVR D}_{i})e^{-\Delta t_{i+1}/\tau}$ $\frac{1}{IVR}\left( G_{i+1} e^{\Delta t_{i+1}/\tau}-G_{i} \right)=D_{i}$ $\sum_{0}^{j-1} D_{i}= \sum_{i=0}^{j-1} \frac{1}{IVR}\left( G_{i+1} e^{\Delta t_{i+1}/\tau}-G_{i} \right)=D$ $\frac{1}{IVR}\sum_{i=1}^{j} G_{i} e^{\Delta t_{i}/\tau}- \frac{1}{IVR}\sum_{i=0}^{j-1} G_{i}=D$ $\frac{1}{IVR}\sum_{i=1}^{j-1} \left( G_{i} e^{\Delta t_{i}/\tau} \right)+ G_{j} e^{\Delta t_{j}/\tau}- \frac{1}{IVR}\sum_{i=1}^{j-1} \left( G_{i} \right)-G_{0}=D$ $\frac{1}{IVR}\sum_{i=1}^{j-1} G_{i}\left( e^{\Delta t_{i}/\tau}-1 \right)+ G_{j}\left( e^{\Delta t_{j}/\tau}-1 \right)=D$ $\frac{1}{IVR}\sum_{i=1}^{j} G_{i}\left( e^{\Delta t_{i}/\tau}-1 \right)=D$ $\sum_{i=1}^{j} c_{i} G_{i}=D$ $\sum_{i=1}^{j} c_{i} G_{i}>\sum_{i=1}^{j} c_{i} G$ $\sum_{i=1}^{j} c_{i} G_{i}$ $\sum_{i=1}^{j} c_{i} G$ $\frac{G}{IVR}\left( e^{\Delta t_{i+1}/\tau}-1 \right)=D_{i}$ $G=\frac{IVR D}{\sum_{i=1}^{j} \left( e^{\Delta t_{i}/\tau}-1 \right)}$ $D_{i}=\left[ \frac{e^{\Delta t_{i+1}/\tau}-1}{\sum_{k=0}^{j-1} \left( e^{\Delta t_{k+1}/\tau}-1 \right)} \right] D$ $\sum_{i=1}^{j} {\Delta t}_{i}=T$ $e^{\Delta t_{i}}-1= {\Delta t_{i}}/\tau+ {\Delta t_{i}^{2}}/\tau+\ldots=T/\left( j \tau\right)+ {T^{2}}/\left( j^{2} \tau^{2} \right)+\ldots$ $\sum_{i=1}^{j} \left( e^{\Delta t_{i}}-1 \right)=T/\left( \tau\right)+ {T^{2}}/\left( j \tau^{2} \right)+\ldots$ $\frac{G}{IVR}=\frac{D}{T/\left( \tau\right)+ {T^{2}}/\left( j \tau^{2} \right)+\ldots}$ $G=\frac{IVR D \tau}{T}.$S3 Appendix. Estimation of bleeds risk

The expected number of bleeds, *B*, over interval *I* between times *t* = *t’* and *t* = *t’’* is

$B=BBR\int_{t'}^{t''} e^{a C_{t}} e^{b {Cat2}_{t}} e^{c {Cat3}_{t}} dt$ (Equation A3.1)

Equation 2 expresses the concentration of factor in the blood at time *t (*i.e., *C_t_*) as a function which depends (through *G_i_*) on the timing and dose of injections. So, by substituting Equation 2 into Equation A3.1, the expected number of bleeds in a given period can be expressed as a function that depends on the prophylaxis regimen

$B=BBR\int_{t'}^{t''} e^{a [E+(G_{P}+{IVR D}_{P})e^{\left( t_{P}-t \right)/\tau}]} e^{b {Cat2}_{t}} e^{c {Cat3}_{t}} dt$

where the subscript *P* refers to the injection preceding time *t* (so *t_P_* is a variable that varies with *t*). If the level of physical activity is constant over the period between *t’* and *t’’*,

$B=BBR e^{b Cat2} e^{c Cat3}\int_{t'}^{t''} e^{a [E+(G_{P}+{IVR D}_{P})e^{\left( t_{P}-t \right)/\tau}]} dt$

For the purpose of calculating the integral we re-scale time so that the clock starts at *t_P_*. Then *t’* and *t’’* become *t’ – t_P_* and *t’’ – tP*. Letting $k= a \left( G_{P}+{IVR D}_{P} \right)$ and $z = e^{-(t-t_{P})/\tau}$

$$\frac{dz}{d(t-t_{P})}= \frac{-1}{\tau} e^{-(t-t_{P})/\tau}$$

$$\frac{dz}{d(t-t_{P})}= \frac{-1}{\tau} z$$

$$d(t-t_{P})= {-\tau dz}/z$$

$B=-\tau BBR e^{b Cat2} e^{c Cat3} e^{a E}\int_{e^{-(t^{'}-t_{P})/\tau}}^{e^{-(t^{''}-t_{P})/\tau}} \left( e^{k z} \right)/z dz$

This can be expressed in terms of the exponential integral,

$$\mathbb{E}_{i}\left\{ kz^{*} \right\} = \int_{z^{*}}^{\infty} \frac{e^{kz}}{z} dz$$

$B= -\tau BBR e^{b Cat2} e^{c Cat3} e^{a E} \left( \mathbb{E}_{i}\left\{ k e^{-(t^{''}-t_{P})/\tau} \right\}- \mathbb{E}_{i}\left\{ k e^{-(t^{'}-t_{P})/\tau} \right\} \right)$ (Equation A3.2)

Writing this equation in terms of the exponential integral is convenient because some software, including Matlab, can calculate the exponential integral. Equation A3.2 provides expressions for the expected number of bleeds in the interval between *t’* and *t’’* following a particular injection.

Equation A3.2 relates the expected number of bleeds in an interval to the time since last injection. This equation is only valid if the level of physical activity is constant over the interval. However the objective here is to determine the expected number of bleeds in a full prophylaxis cycle, and a full prophylaxis cycle may consist of multiple injections and changing levels of physical activity. So, to calculate the expected number of bleeds in a full prophylaxis cycle, the cycle is divided into a series of intervals. A new interval, *I*, commences when there is an injection or when there is a change in physical activity category. The expected number of bleeds in a full cycle of prophylaxis, *B_cycle_*, is the sum of the expected number of bleeds across all intervals in the cycle

$B_{cycle}= \sum_{I} \left[ -\tau BBR e^{b {Cat2}_{I}} e^{c {Cat3}_{I}} e^{a E} \left( \mathbb{E}_{i}\left\{ k e^{-(t_{I}^{'}-t_{P})/\tau} \right\}- \mathbb{E}_{i}\left\{ k e^{-(t_{I}^{''}-t_{P})/\tau} \right\} \right) \right]$

(Equation A3.3)

The incidence rate ratio for the cycle is *IRR* = *B_cycle_ / (T BBR)*. By substituting Equation A3.3 into this expression it can be seen that

$IRR= \sum_{I} \left[ -\tau e^{b {Cat2}_{I}} e^{c {Cat3}_{I}} e^{a E} \left( \mathbb{E}_{i}\left\{ k e^{-(t_{I}^{'}-t_{P})/\tau} \right\}- \mathbb{E}_{i}\left\{ k e^{-(t_{I}^{''}-t_{P})/\tau} \right\} \right) \right] / T$ (Equation A3.4)

When using a risk-based approach to optimizing prophylaxis we might choose to minimize *IRR*. Alternatively, we could convert *B_cycle_*, the expected number of bleeds in a prophylaxis cycle, to an annual bleeds rate, *ABR*. Minimizing the *ABR* is equivalent to minimizing *the IRR*, but it is easier to appreciate the magnitude of the effects of different prophylaxis regimens when minimizing *ABR* rather than *IRR*. The annual bleed rate, *ABR*, is *B_cycle_ × (1 year / T)*. Substituting Equation A3.3

$ABR= \sum_{I} \left[ -\tau BBR e^{b {Cat2}_{I}} e^{c {Cat3}_{I}} e^{a E} \left( \mathbb{E}_{i}\left\{ k e^{-(t_{I}^{'}-t_{P})/\tau} \right\}- \mathbb{E}_{i}\left\{ k e^{-(t_{I}^{''}-t_{P})/\tau} \right\} \right) \right] \times1 year / T$

Calculation of the *ABR* requires an estimate of the *BBR*. Note that *BBR* is the *baseline* bleed rate, not the observed bleed rate. The *BBR* cannot be directly observed in people who engage in category 2 or category 3 physical activity, or who have any endogenous factor VIII, or are on prophylaxis. However the *BBR* could be estimated using information about (a) the timing and dose of injections in a typical prophylaxis regimen, (b) a typical pattern of physical activity, and (c) the observed number of bleeds in a prophylaxis cycle (equal to the annualized number of bleeds *× T / 1 year*). These data could be collected retrospectively. Then the *BBR* could be estimated by solving Equation A.3 for *BBR*

${BBR}_{[obs]}= B_{cycle [obs]} / \sum_{I} \left[ -\tau e^{b {Cat2}_{I}} e^{c {Cat3}_{I}} e^{a E} \left( \mathbb{E}_{i}\left\{ k e^{-(t_{I[obs]}^{'}-t_{P[obs]})/\tau} \right\}- \mathbb{E}_{i}\left\{ k e^{-(t_{I[obs]}^{''}-t_{P[obs]})/\tau} \right\} \right) \right]$

The subscript *[obs]* indicates the values for *B_cycle_* and for the patterns of activity and prophylaxis regimen are those that have been observed.
