# Supplementary material for: Correction: Optimization of prophylaxis for hemophilia A
Source: PLoS One. 2018 Apr 30;13(4):e0196695. doi: 10.1371/journal.pone.0196695 (PMC5927422; doi:10.1371/journal.pone.0196695)
Supplement: S1 Appendix — (PDF) [file pone.0196695.s001.pdf]

S1 Appendix. Residual plasma factor VIII concentration

Rowland and Tozer [14; p554] provide equations for steady state concentrations in cycles with varying intervals between injections. They also provide more general equations for arbitrary doses and intervals by assuming that the residual concentration after four half-lives is negligible. Here we provide equations to determine the steady state in a recurring cycle with varying doses and intervals. The approach does not ignore the residual concentration from any previous injections.

At steady state, each trough concentration must be equal to the corresponding trough concentration in the next cycle. So:

if there is just *one* injection in a cycle,

$$E + G_1 = E + (G_1 + IVR\,D_1)\,e^{-T/\tau},$$

if there are *two* injections in a cycle,

$$E + G_1 = E + \left[(G_1 + IVR\,D_1)\,e^{(t_1-t_2)/\tau} + IVR\,D_2\right] e^{(t_2-T-t_1)/\tau},$$

if there are *three* injections in a cycle,

$$E + G_1 = E + \left\{\left[(G_1 + IVR\,D_1)\,e^{(t_1-t_2)/\tau} + IVR\,D_2\right] e^{(t_2-t_3)/\tau} + IVR\,D_3\right\} e^{(t_3-T-t_1)/\tau},$$

and so on. Thus, for three injections,

$$E + G_1 = E + \left(IVR\,D_1\,e^{(-T)/\tau} + IVR\,D_2\,e^{(t_2-T-t_1)/\tau} + IVR\,D_3\,e^{(t_3-T-t_1)/\tau}\right) / \left(1 - e^{(-T)/\tau}\right)$$

More generally, at steady state in a cycle of *j* injections, the trough preceding the first injection in the cycle is

$$E + G_1 = E + \left(\sum_{i=1}^{j} IVR\,D_i\,e^{(t_i-T-t_1)/\tau}\right)/\left(1 - e^{-T/\tau}\right)$$

As it is arbitrary which injection starts the cycle, and therefore which is indexed as injection 1, an even more general expression is

$$E + G_n = E + \left(\sum_{i=1}^{j} IVR\ D_i\ e^{(t_i - T - t_n)/\tau}\right) / \left(1 - e^{-T/\tau}\right)$$

provided t_i is replaced with $T + t_i$ when $i < n$.

References

1. Rowland M, Tozer TN. Clinical Pharmacokinetics and Pharmacodynamics: Concepts and Applications. 4th ed. Baltimore: Williams & Wilkins; 2011.
