# Supplementary material for: Correction: Optimization of prophylaxis for hemophilia A
Source: PLoS One. 2018 Apr 30;13(4):e0196695. doi: 10.1371/journal.pone.0196695 (PMC5927422; doi:10.1371/journal.pone.0196695)
Supplement: S2 Appendix — (PDF) [file pone.0196695.s002.pdf]

S2 Appendix. Prophylaxis regimens that maximize the lowest factor VIII concentration

When seeking to identify prophylaxis regimens that maximize the lowest factor VIII concentration we can ignore the (constant) endogenous factor concentration, *E*, by setting it to 0. Then trough concentrations satisfy the relation

$$G_{i+1} = (G_i + IVR\ D_i)e^{-\Delta t_{i+1}/\tau}.$$

where t_j is the end of the cycle and $\Delta t_{i+1} = t_{i+1} - t_i$. Rearranging yields

$$\frac{1}{IVR}\left(G_{i+1}\, e^{\Delta t_{i+1}/\tau} - G_i\right) = D_i \qquad \text{(Equation A2.1)}$$

and summing the D_i to obtain the total dose in a cycle gives

$$\sum_0^{j-1} D_i = \sum_{i=0}^{j-1} \frac{1}{IVR}\left(G_{i+1}\, e^{\Delta t_{i+1}/\tau} - G_i\right) = D$$

$$\frac{1}{IVR}\sum_{i=1}^{j} G_i\, e^{\Delta t_i/\tau} - \frac{1}{IVR}\sum_{i=0}^{j-1} G_i = D$$

From the former summation pull out the $i = j$ term and from the latter pull out the $i = 0$ term, so

$$\frac{1}{IVR}\sum_{i=1}^{j-1}\left(G_i\, e^{\Delta t_i/\tau}\right) + G_j\, e^{\Delta t_j/\tau} - \frac{1}{IVR}\sum_{i=1}^{j-1}(G_i) - G_0 = D$$

In the steady state $G_0 = G_j$, so

$$\frac{1}{IVR}\sum_{i=1}^{j-1} G_i\left(e^{\Delta t_i/\tau} - 1\right) + G_j\left(e^{\Delta t_j/\tau} - 1\right) = D$$

$$\frac{1}{IVR}\sum_{i=1}^{j} G_i\left(e^{\Delta t_i/\tau} - 1\right) = D$$

Let $c_i = \left(e^{\Delta t_i/\tau} - 1\right)/IVR$. Then

$$\sum_{i=1}^{j} c_i\, G_i = D \qquad \text{(Equation A2.2)}$$

Consider the scenario in which the total dose and the injection schedule are fixed but the total dose can be shared between injections in any way. Note that fixing the injection schedule fixes the values of c_i. Equation A2.2 shows that if there exists a regimen that shares doses across injections in a way which makes all of the troughs in a cycle equal (i.e., which makes all $G_i = G$) that regimen is the regimen that maximizes the lowest factor VIII concentration. This must be true because it is not possible that all of the troughs $G_i > G$; otherwise, if all $G_i > G$, then $\sum_{i=1}^{j} c_i\, G_i > \sum_{i=1}^{j} c_i\, G$, which is inconsistent with Equation A2.2 because it is then not possible that both $\sum_{i=1}^{j} c_i\, G_i$ and $\sum_{i=1}^{j} c_i\, G$ both equal D. Thus, for any given injection schedule, the regimen which maximizes the lowest factor VIII concentration is the regimen that makes the troughs equal, if such a regimen exists.

A regimen that makes the troughs equal always exists. This can be demonstrated by making all the trough values $G_i = G$. Then, from Equation A2.1

$$\frac{G}{IVR}\left(e^{\Delta t_{i+1}/\tau} - 1\right) = D_i \qquad \text{(Equation A2.3)}$$

Summing across i gives

$$G = \frac{IVR\ D}{\sum_{i=1}^{j}\left(e^{\Delta t_i/\tau} - 1\right)} \qquad \text{(Equation A2.4)}$$

This is the trough concentration that is attained when all of the troughs are equal, so it is also the trough concentration when the regimen is optimal (in the sense of maximizing the lowest factor VIII concentration) for a given injection schedule. Substituting Equation A2.3 into A2.4 gives

$$D_i = \left[\frac{e^{\Delta t_{i+1}/\tau} - 1}{\sum_{k=0}^{j-1}\left(e^{\Delta t_{k+1}/\tau} - 1\right)}\right] D$$

The quantity in the square brackets is the proportion of D that is D_i. It has a value between 0 and 1, so D_i has a value between 0 and D. To the extent that it is possible to give injections with doses of anywhere between 0 and D, this implies that there always exists a regimen that makes the troughs equal, so there is always a regimen that maximizes the lowest factor VIII concentration for any injection schedule. In practice it may be difficult to administer that optimal regimen.

The preceding part of this appendix shows that the optimal regimen *for a given injection schedule* is a regimen which shares the total dose across injections in a way that makes all trough concentrations equal. Now we allow the injection schedule to vary and we ask which of all possible regimens with dose *D* and number of injections *j* maximizes the lowest factor VIII concentration. We can conceive of the injection schedule (the set of Δt_i) as an independent variable and the trough concentration $G = G(\Delta t_i)$ to be a function of the injection schedule (Equation A2.4) subject to the constraint that $\sum_{i=1}^{j} \Delta t_i = T$.

The method of Lagrangian multipliers shows that *G* is maximized when $\partial G/\partial \Delta t_i = \lambda$, where λ is the Lagrangian multiplier. Rearranging gives $\partial \Delta t_i = \partial G/\lambda$. Note that the expression for $\partial \Delta t_i$ does not depend on *i*. This shows that when *G* is maximized, Δt_i is constant (i.e., all Δt_i are equal). So, of all possible injection schedules, the lowest factor VIII concentration is maximized when the intervals between injections are equal. We have already seen that if the lowest factor VIII concentration is to be maximized the trough concentrations must be equal. And if both the intervals between injections are equal and the trough levels are equal, the doses of each of the injections must also be equal. So, of all possible injection regimens, the lowest factor VIII concentration is maximized when the intervals between injections are equal and the doses of the injections are equal.

In the preceding paragraph we fixed the number of doses. What is the optimal number of doses? Since the optimal schedule has equal intervals between injections, $\Delta t_i = T/j$. As the number of doses goes to infinity, $\Delta t_i \rightarrow 0$, so $e^{\Delta t_i} - 1 = \Delta t_i/\tau + \Delta t_i^2/\tau + \cdots = T/(j\,\tau) + T^2/(j^2\,\tau^2) + \cdots$.

As a consequence,

$$\sum_{i=1}^{j} \left(e^{\Delta t_i} - 1\right) = T/(\tau) + T^2/(j\,\tau^2) + \cdots,$$

and

$$\frac{G}{IVR} = \frac{D}{T/(\tau) + T^2/(j\,\tau^2) + \ldots}$$

As $j \rightarrow \infty$, $T/(\tau) + T^2/(j\,\tau^2) + \cdots$ approaches its minimum, which is $T/(\tau)$. As a consequence, G approaches its maximum at $G = \frac{IVR\;D\;\tau}{T}$. This is the steady state concentration when a continuous infusion is given.
